# Research priorities for managing the impacts and dependencies of business upon food, energy, water and the environment

**DOI:** 10.1007/s11625-016-0402-4

**Published:** 2016-10-07

**Authors:** Jonathan M. H. Green, Gemma R. Cranston, William J. Sutherland, Hannah R. Tranter, Sarah J. Bell, Tim G. Benton, Eva Blixt, Colm Bowe, Sarah Broadley, Andrew Brown, Chris Brown, Neil Burns, David Butler, Hannah Collins, Helen Crowley, Justin DeKoszmovszky, Les G. Firbank, Brett Fulford, Toby A. Gardner, Rosemary S. Hails, Sharla Halvorson, Michael Jack, Ben Kerrison, Lenny S. C. Koh, Steven C. Lang, Emily J. McKenzie, Pablo Monsivais, Timothy O’Riordan, Jeremy Osborn, Stephen Oswald, Emma Price Thomas, David Raffaelli, Belinda Reyers, Jagjit S. Srai, Bernardo B. N. Strassburg, David Webster, Ruth Welters, Gail Whiteman, James Wilsdon, Bhaskar Vira

**Affiliations:** 1 Department of Geography and University of Cambridge Conservation Research Institute, Downing Site, Cambridge, CB2 3EN UK; 2 University of Cambridge Institute for Sustainability Leadership, 1 Trumpington Street, Cambridge, CB2 1QA UK; 3 Stockholm Environment Institute, Environment Building, University of York, Wentworth Way, York, YO10 5NG UK; 4 Conservation Science Group, Department of Zoology, University of Cambridge, The David Attenborough Building, Pembroke Street, Cambridge, CB2 3QZ UK; 5 Openfield, Honey Pot Lane, Colsterworth, Grantham, Lincs, NG33 5LY UK; 6 UK Global Food Security Programme, BBSRC, Polaris House, North Star Avenue, Swindon, SN2 1UH UK; 7 School of Biology, University of Leeds, Leeds, LS2 9JT UK; 8 Swedish Steel Association (Jernkontoret), Kungsträdgårdsgatan 10, Box 1721, 111 87 Stockholm, Sweden; 9 Environment Research Group, School of Natural Sciences and Psychology, Liverpool John Moores University, Byrom Street Campus, Byrom Street, Liverpool, L3 3AF UK; 10 Saint Gobain Building Distribution UK, Merchant House, Binley Business Park, Binley, Coventry, CV3 2TT UK; 11 Anglian Water, Lancaster House, Lancaster Way, Ermine Business Park, Huntingdon, PE29 6YJ UK; 12 Asda, Asda House, Southbank, Great Wilson Street, Leeds, LS11 5AD UK; 13 Mondi Group, Building 1, 1st Floor, Aviator Park, Station Road, Addlestone, KT15 2PG UK; 14 Centre for Water Systems, College of Engineering, Mathematics and Physical Sciences, University of Exeter, Harrison Building, Streatham Campus, North Park Road, Exeter, EX4 4QF UK; 15 Economic and Social Research Council, Polaris House, North Star Avenue, Swindon, SN2 1UJ UK; 16 Kering, 10 Avenue Hoche, 75381 Paris Cedex 08, France; 17 Ovo Energy, 1 Rivergate Temple Quay, Bristol, BS1 6ED UK; 18 GlaxoSmithKline, 980 Great West Road, Brentford, TW8 9GS UK; 19 Stockholm Environment Institute, Linnégatan 87D, Box 24218, 104 51 Stockholm, Sweden; 20 Centre for Ecology and Hydrology, Maclean Building, Benson Lane, Crowmarsh Gifford, Wallingford, OX10 8BB UK; 21 Global Headquarters, Nestlé S.A, Avenue Nestlé 55, 1800 Vevey, Switzerland; 22 HSBC Bank, 8 Canada Square, Canary Wharf, London, E14 5HQ UK; 23 EDF Energy, Cardinal Place, 80 Victoria Street, London, SW1E 5JL UK; 24 Advanced Resource Efficiency Centre and Management School, University of Sheffield, Conduit Road, Sheffield, S10 1FL UK; 25 Ernst and Young LLP, 1 More London Place, London, SE 2AF UK; 26 WWF and the Natural Capital Project, The Living Planet Centre, Rufford House, Brewery Road, Woking, Surrey, GU21 4LL UK; 27 UKCRC Centre for Diet and Activity Research (CEDAR), MRC Epidemiology Unit, University of Cambridge School of Clinical Medicine, Institute of Metabolic Science, Box 285, Cambridge Biomedical Campus, Cambridge, CB2 0QQ UK; 28 School of Environmental Sciences, University of East Anglia, Norwich, NR4 7TJ UK; 29 Bidvest Fresh Limited, Cedar way, Camley Street, London, N1C 4PD UK; 30 ArcelorMittal, Berkeley Square House, 7th Floor, Berkeley Square, London, W1J 6DA UK; 31 BESS Directorate, Environment, University of York, Heslington, York, YO10 5DD UK; 32 Stockholm Resilience Centre, Stockholm University, Kräftriket 2B, SE-106 19 Stockholm, Sweden; 33 Institute for Manufacturing, Department of Engineering, University of Cambridge, Charles Babbage Road, Cambridge, CB3 0ES UK; 34 International Institute for Sustainability & Department of Geography and the Environment, Pontifical Catholic University of Rio de Janerio, Estrada Dona Castorina 124, Horto, Rio de Janeiro, Brazil; 35 Jordans and Ryvita, Market Garden Road, Biggleswade, SG18 8QB UK; 36 Pentland Centre for Sustainability in Business, Lancaster University, Bailrigg, Lancaster, LA1 4YX UK; 37 SPRU-Science Policy Research Unit, University of Sussex, Brighton, BN1 9SL UK

**Keywords:** Corporate sustainability, Nexus interactions, Environment, Food security, Energy security, Water security

## Abstract

Delivering access to sufficient food, energy and water resources to ensure human wellbeing is a major concern for governments worldwide. However, it is crucial to account for the ‘nexus’ of interactions between these natural resources and the consequent implications for human wellbeing. The private sector has a critical role in driving positive change towards more sustainable nexus management and could reap considerable benefits from collaboration with researchers to devise solutions to some of the foremost sustainability challenges of today. Yet opportunities are missed because the private sector is rarely involved in the formulation of deliverable research priorities. We convened senior research scientists and influential business leaders to collaboratively identify the top forty questions that, if answered, would best help companies understand and manage their food-energy-water-environment nexus dependencies and impacts. Codification of the top order nexus themes highlighted research priorities around development of pragmatic yet credible tools that allow businesses to incorporate nexus interactions into their decision-making; demonstration of the business case for more sustainable nexus management; identification of the most effective levers for behaviour change; and understanding incentives or circumstances that allow individuals and businesses to take a leadership stance. Greater investment in the complex but productive relations between the private sector and research community will create deeper and more meaningful collaboration and cooperation.

## Introduction

Delivering access to sufficient food, energy and water resources to ensure human wellbeing, both now and in the future, is a major concern for governments worldwide (Guerry et al. 2015). These are emphasised in many of the newly adopted sustainable development goals of the United Nations, and the targets for the 2030 development agenda (United Nations 2014). Here, we describe a process to bring together the research and business communities in an exercise to devise a co-produced list of urgent but deliverable research priorities for more sustainable management of food, energy, water and the environment. These priorities can be used by funding agencies and businesses themselves to target investment towards policy- and business-relevant research.

A serious challenge lies in the provision and distribution of sufficient food, water and energy resources to supply a global population that is increasing in size and in levels of consumption. Central to this problem is the need to understand and account for the manner in which food, energy, water and the environment interact, and the implications of these interactions for human wellbeing. Both policy and research communities increasingly refer to this interconnected milieu as the ‘nexus’ (Beddington 2009; Vira 2015). This nexus of complex interactions, which will include unpredictable step changes and reinforcing responses (Halbe et al. 2015), is poorly understood yet can have profound consequences for human wellbeing, poverty and inequality. Human food production systems, for instance, are heavily dependent upon energy for fertilisers, water for irrigation, and nature’s functions for cycling nutrients and pollinating crops. Altering the availability or demand of one can have severe but unexpected repercussions for the others. Continued depletion and degradation of the natural environment further compromises its ability to meet predicted increases in demand for food, energy and water and presents a very real threat to economic prosperity and to livelihoods, which are rendered ever more vulnerable in many parts of the world (Vira 2015). These uncertainties and vulnerabilities present a business case that is both pragmatic, because food, energy and water availability cannot be guaranteed, and moral, as recently outline by the Pope in his notable encyclical (Catholic Church 2015). For many businesses in the nexus mix, the pragmatic and moral combine to create a fresh cooperative business perspective. The inequalities around basic aspects of wellbeing such as nutrition, health, sanitation and security, lie at the heart of concerns to understand how the complex nexus of interactions can be better managed.

Although governments have a critical role in devising and implementing policy to minimise the potentially devastating impacts nexus crises, it is increasingly recognised that the private sector has a vital role to play (Guerry et al. 2015; Wales 2014). In a comprehensive analysis of businesses responses to planetary boundaries, Whiteman et al. (2012), show that in general businesses have not addressed either water scarcity or biodiversity vulnerability to any consistent extent. Environmental risks are generally perceived to manifest over medium to long term timescales and for business, pushed to look at quarterly reporting; these risks are seen as important, but not yet requiring immediate action (WEF 2016). However, floods, storms, conflict over scarce resources and resultant insecurity and potential loss of access to raw materials have already begun to collapse the timescales of nexus risks, bringing them within conventional planning schedules for business. Clearly, the private sector is not a homogenous group of business with respect to exposure to nexus risk and capacity or commitment to deal with it. Increasing awareness and regulation around environmental issues has, however, helped catalyse private sector actors to address nexus governance challenges (Cranston et al. 2015). An improved sustainability record that addresses nexus risks can directly benefit business though decreased procurement costs (through efficient use of scarce resources), lessened risk (for example by anticipating regulatory demands or preventing degradation of required natural resources), and enhanced organisational reputation or market differentiation to increase competitiveness (Cranston et al. 2015). Businesses recognise that there are gaps in their approaches to improving their sustainability practice. These tend to be around lack of understanding, missing business-relevant evidence, disparate policy and the need for greater internal engagement (Andrade-Afonso and Cranston 2013). It was hypothesised that these themes would be again identified by business when focusing specifically on nexus issues. However, actors beyond the private sector have different perspectives and motivations, so when research needs are co-designed by a multi-stakeholder group it is less easy to anticipate which concerns will emerge as priorities.

The private sector clearly has the financial and human resources to act and help shape global responses to nexus challenges: fifty-eight per cent of the top 150 economic entities in the world are corporations rather than countries (Kareiva et al. 2015; Keys et al. 2013). Moreover, corporations are often operating at the nexus of interactions—for instance by ensuring that supply chains are resilient and able to continue to provide food, energy or water in the face of external shocks (Whiteman et al. 2012). The private sector also often wields considerable influence over decisions affecting the provision of food, energy and water. Anchored in practical implementation, the private sector perspective, therefore, has a critical role in dialogue to devise research agendas for a more sustainable future for the continued supply of food, energy and water. There is also an increasing trend for companies to be held to account for both financial and non-financial performance; for example, within the growing Sustainable Stock Exchange Initiative (SSE 2014) and recent years have seen increased numbers of businesses taking action to ensure that their supply chains are more sustainable and more resilient, their licence to operate is secure and their risks are adequately managed (Cranston et al. 2015; Maxwell et al. 2014). In one example, a group of companies, recognising the risk that poor water security posed for their businesses, came together to develop a collaborative solution (Ya He and Cranston 2014). The companies came to the problem of water security from different viewpoints, though grounded in the need to improve business operations, secure supply chains and reduce risks. They ranged from the provision of food for farmers and retailers to the impacts upon the environment that their operations were having. Clear interdependencies were identified by the different stakeholders and working collectively across sectors delivered more effective water strategies and finance mechanisms that recognised the value of water to the interdependent nexus elements across different sectors.

Whilst there are numerous examples of cross-sectoral coalitions to identify sustainability targets or actions (e.g., United Nations 2014; World Business Council for Sustainable Development 2014; CISL 2016), the transition of knowledge from the outputs of academic research through to changes in business practice remains difficult to achieve (Knight et al. 2008; Lang et al. 2012; Pohl et al. 2010). One approach is to bring together researchers and industry partners to devise solutions around specific problems, adopting a structured process to generate a shared view of future research challenges and priorities (Sugiyama et al. 2016). Examples of such an expert-based approach to identify research priorities are found in a wide range of disciplines: from pollinator conservation (Dicks et al. 2013) to communication of risk (Chess et al. 1995) and, especially, medical science (Baulac and Pitkänen 2009; El-Jardali et al. 2010; Deane et al. 2014). These research priorities are used by policy-makers and research councils; for example in developing Defra’s UK Marine Science Strategy (Sutherland et al. 2006), and for developing the priorities of the Global Food Security Programme (Dicks et al. 2013; Pretty et al. 2010). It is crucial that the complexities and limitations faced by business are accounted for early on in the research process such that knowledge generated is relevant, accessible and actionable and, as a result, more easily incorporated into business practice and government policy (El-Jardali et al. 2010; Lang et al. 2012; Wiek et al. 2012).

## Materials and methods

The process for identifying and ranking the most important research questions is described in detail in Sutherland et al. (2011) and hinges upon three key principles: (1) questions should be solicited from a diverse group, representing different sectors, disciplines and geographies, (2) the credibility of the workshop attendees is crucial—they must have, and be recognised as having, the knowledge base and positional experience to be able to refine and prioritise questions and (3) the process must be democratic, transparent and accountable (Sutherland and Burgmann 2015)—both within the group during the prioritisation process, and subsequently as the results are disseminated.

### Gathering questions

Questions were solicited between 5 March and 31 July 2015 from a diverse group of people through workshops, webinars, presentations, social media, targeted email outreach and opportunistic promotion, such as through email signatures and in discussions with colleagues (Table 1). Individuals were invited to submit research questions in response to the request:

**Table 1:** Number of questions submitted by each sector for each outreach event. In brackets is the number of participants for the event (or, in the case of targeted outreach, recipients)

	Webinars^a^	Workshops^b^	Presentations^c^	Social media^d^	Targeted outreach^e^	General promotion^f^	Total
Number of participants	(33)	*(>114)*	*(269)*		*(>120)*		
Researchers	41	116	3	22	39	45	266
Non-governmental organisation		22		12	3	6	43
Civil Service		9				7	16
Private sector	25	196	61	32	39	30	383
>Agric., Forestry, Fishing & Extractives	4	24		1	11	1	41
>Finance, Legal, Insurance & Investment		9	2	5	7		27
>Consultancy, Media & IT	21	21	11	17	5	11	86
>Food & Beverage		19			3	1	23
>Construction, Manufacturing & Consumer Goods		34	3	8		5	50
>Retail		70			1	3	74
>Utilities & Waste Management		7	27	1	5	5	45
>Other		12	18		7		37
Not specified		8		6			14
Total number of questions submitted	66	351	64	72	81	88	722

Participant numbers in italics are estimates

^a^One webinar with CISL alumni and two webinars with the Nexus Network

^b^Eight workshops were carried out. Workshop is defined as an event during which some time is specifically dedicated to interactive discussion. Workshops were hosted for staff at Asda, members of the University of Cambridge Conservation Research Institute, academic staff at Lancaster Environment Centre and Lancaster University Management School, attendees at a business and academic engagement event hosted by CISL, students on two teaching programmes at CISL, staff at CISL staff and staff at the World Wildlife Fund

^c^Four presentations were given to solicit questions. The term ‘Presentations’ refers to events during which Nexus2020 was introduced and people could submit questions at their leisure. Nexus2020 was presented at the Institute of Water 2015 annual conference, Global Soil Week Conference 2015 at the Institute for Advanced Sustainability Studies and the Nexus Network Methods Conference

^d^Social media included a Twitter chat hosted by Farming First, a Virtual Learning Environment, where it was posted for four different CISL student cohorts, and LinkedIn, where it was posted on four groups: 2degrees, Cambridge Network, Cambridge Sustainability Network and Sustainability professionals

^e^Targeted outreach through, for example, emails, invitations and leaflet promotion at CISL events

^f^General promotion through, for example, email signatures, promotion on CISL and Nexus Network webpages, newsletters and word of mouth

*“What are the most important questions around business practice that, if answered, could help companies manage their dependencies and impacts upon food, energy, water and the environment?”*

Contributors were advised that questions should be specific (rather than a general topic for research) and that they should be formulated as a question that might result in a research process that could generate an answer over the next 5 years. Questions should be either useful for, or relevant to, business. Not all research questions were anticipated to directly result in changes to business practice. Some, when answered, might provide evidence to help regulatory bodies or consumers encourage or drive change in business behaviour. Contributed questions also, therefore, included those that help us to understand aspects of regulation, policy or consumption choices that affect business practice and sustainability.

Various methods were used to prompt participants to produce researchable questions, including the sharing of example questions to all participants, speaker-based events, peer-to-peer discussions, and workshops in which facilitators worked with participants from problem statements through to specific research questions. To avoid bias formed from sampling a group with a narrow range of geographic interests, we ensured a broad range of international experience amongst contributors and workshop attendees. In total 722 questions were submitted by at least 238 individuals from at least 152 institutions or companies (some were submitted anonymously). Sixteen questions were excluded prior to the first round of voting for being incomplete or clearly unrelated to the topic. The nature of nexus thinking is that any one element cannot be researched or acted upon in isolation. Forty-eight per cent of questions did not explicitly mention food, energy, water, or the environment but, of those that did, the majority focused on just one (thirty-one per cent of all questions), followed by two (eleven per cent), three (five per cent) or all four elements (five per cent).

### Prioritisation

Once question submission was closed, three of the authors (JMHG, GRC, HRT) independently grouped the questions into research areas, before coming together to agree on twelve broad categories, which each received approximately sixty questions: (1) Consumption, consumer behaviour and demand-side issues; (2) Measuring, reporting and transparency; (3) Technological solutions, instruments and innovation; (4) Policy, regulation and governance; (5) Awareness, education and communications; (6) Ecosystem services, valuation and externalities; (7) Resource efficiency, waste and the circular economy; (8) Collaboration, stakeholder engagement and supply chain influence; (9) Decision making, mutual benefits and trade-offs; (10) Forecasting, future scenarios and risk; (11) Land use, practical applications and direct impacts; and (12) Change agents, financial systems/incentives and leverage points. These groupings are useful for helping participants compare similar questions by ensuring that questions that overlap or complement one another are placed together as they complete the prioritisation exercise.

After categorisation, the subdivided list was emailed to workshop participants for an initial vote, for which individuals identified the most important four to six questions from those categories within their expertise. Twenty-three participants voted in all twelve categories, while eight participants voted on a subset of categories. These votes were then used to rank the questions within each category. Following this first round of voting, twenty-three participants from the research community and seventeen from the business community came together at a two-day workshop held in Cambridge, UK, during September 2015.

During the second round of voting each category was discussed in its own session, attended by approximately ten participants. Each session had a chairperson and a facilitator chosen from the workshop participants and a note taker. If there was disagreement within the group, the group was polled and a majority decision was taken. During each session, the questions were discussed to identify, within each of the groups, a maximum of twenty-four questions to go through to the next stage. With the consensus of the group, questions could be edited, split, added or reformulated to improve them. These were allocated to ‘gold’, ‘silver’ and ‘bronze’ (eight in each) according to their relative merit, as judged by being important, answerable under the agreed research conditions, and relevant to business (Table 2; see Sutherland et al. 2013). The categories allowed the smaller group to identify their top priorities, whilst also allowing a larger subset of prioritised questions into the subsequent round for review by the wider group. This process was informed, but not restricted, by the votes received in the first round. Therefore, some questions that had received few votes in the initial stage emerged from this second round with gold status, whilst others with many votes were dropped, following discussion, in favour of other questions.

**Table 2:**
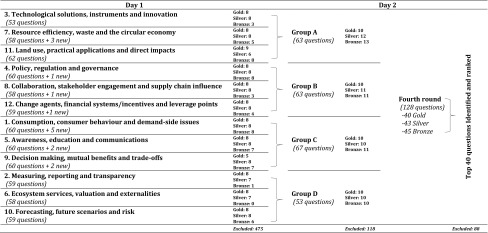
Workflow for the prioritisation workshop

Following the first round of voting, participants were convened at a two-day workshop to conduct three further rounds of voting, during which the 40 most important questions were identified from an initial long-list of 706 questions. In addition, 15 new questions were formed based on the discussions of day one and these were also fed into the process

Reasons for eliminating questions from inclusion in the next round were recorded (Table 3). Of those questions with a reason specified, many did not meet the qualifying criteria: eleven per cent of questions were excluded on the basis of being irrelevant to the topic; ten per cent because the subject was deemed by consensus to be sufficiently well understood, such that further research would do little to help companies better manage their dependencies and impacts upon food, energy, water and the environment (or receiving sufficient attention under currently funded projects); and four per cent for being unanswerable due to timescale or budget limitations or because the question was considered unsuited to a research project (Table 3). Two per cent of questions were not included because they were considered to be too specific. Some important but sector-specific challenges will therefore not be reflected in the final priority list. There was a high level of agreement around the knowledge gaps that most urgently need to be filled through future research amongst question contributors, evidenced by the fact that repetition caused twenty-four per cent of the questions in the initial long-list of questions to be excluded from subsequent stages. The reason for excluding questions was not specified in thirty-one per cent of cases. This included questions that received no votes in the first round and, following review, were not defended by any of the attendees during the second round, and thereby deemed by consensus not to be a priority.

**Table 3:** Specified reasons for dropping questions during the second round of voting. When workshop participants specified the reason for dropping a question from the priority list on day one of the workshop it was recorded and is reported below

Group	Not relevant (%)	Already answered (%)	Repetition (%)	Too generic (%)	Can’t be answered (%)	Too specific (%)	Low priority (%)	Not specified (%)	Total Rejected
1. Consumption, consumer behaviour and demand-side issues	–	4	9	38	2	4	–	42	41
2. Measuring, reporting and transparency	5	17	43	12	10	10	5	–	43
3. Technological solutions, instruments and innovation	9	21	6	9	3	–	3	48	34
4. Policy, regulation and governance	8	–	14	3	3	–	8	64	37
5. Awareness, education and communications	15	8	23	21	10	3	3	18	39
6. Ecosystem services, valuation and externalities	5	24	40	10	5	–	–	17	43
7. Resource efficiency, waste and the circular economy	3	–	21	18	–	–	–	58	40
8. Collaboration, stakeholder engagement and supply chain influence	10	2	27	5	–	2	5	49	40
9. Decision making, mutual benefits and trade-offs	3	5	35	13	8	–	5	33	42
10. Forecasting, future scenarios and risk	37	14	23	11	–	3	–	11	37
11. Land use, practical applications and direct impacts	21	13	15	–	3	–	10	38	39
12. Change agents, financial systems/incentives and leverage points	17	7	27	32	2	–	12	2	40
Total	11	10	24	15	4	2	4	31	475

On the second day the twelve groups were merged to form four groups for the third round of voting, in which questions were initially ranked by their gold, silver or bronze classification of the previous day (Table 2). Each group was attended by approximately twenty people. The list was reviewed in detail again and the top questions (approximately thirty per session) were chosen, initially based on the top twenty-four gold questions that fed in from the three sessions of the previous day, but allowing each of the silver and bronze questions to be individually assessed on their merits for possible inclusion. Once the top questions were identified, they were categorised gold, silver or bronze (ten for each) before being reviewed and passed into the fourth and final round of voting. The final session convened the entire group to identify the top forty questions from a list of 128 that had come through from the third round of voting. Each participant also voted for the fifteen from this list that they considered most urgently in need of attention. This allows the subsequent classification of a ‘platinum’ subset of questions that can be used to identify, from amongst a longer list of important questions, those that have the most pressing needs, given limited research capacity and funds. Following the workshop, the forty prioritised questions were edited for clarity and agreed with all workshop participants.

This whole process was collegiate. Businesses in different aspects of the Nexus were able to identify their concerns and knowledge gaps, but the manner of the preparation and the design of the workshop enabled a collective approach. The twelve item template of key themes which emerged from the first round enabled focus for the subsequent stages. The outcome was a sense of commitment built on very differing but supportive expertise and experience.

## Results and discussion

Each of the twelve initial categories from the first and second rounds of voting was represented at least once in the final list. On average, the forty prioritised questions (Table 4) received a median of four votes in the first round of voting (range = 0–10), while the median across all submitted questions was two votes (range = 0–12, *n* = 706). Overall, there was a reasonable balance of “questions for business” and “questions about business” (private sector sustainability from the perspective of others, such as how to effectively regulate or incentivise business). The questions within these categories can be clustered around emergent themes, which are discussed below.

**Table 4:** The top 40 questions prioritised during the two-day workshop are listed alongside the original category assigned to them (see Table 2). Participants assessed the questions’ relative priority and they are presented in descending order of the number of votes given

Q#	Question	Category	Votes
(1)	What are the most effective ways to incorporate social considerations into nexus decision-making processes (that allow companies to simultaneously manage their own risks whilst also eliminating risks resulting from their activities on livelihoods, land and water security of marginalised or vulnerable groups) and what are their limitations?	Group 6	18
(2)	What are the critical nexus trade-offs, hotspots and risk scenarios and what are the implications of these for business and society?	Group 2	16
(3)	What is the relative impact of information, pricing, nudging and taxation on businesses and consumers, and how do these approaches differ in terms of their effectiveness, implications for equity and acceptability?	Group 1	15
(4)	What are the most effective ways to incentivise or regulate businesses to value their dependencies and their impacts on ecosystem services (including consideration of the potential insurance value of biodiversity)?	Group 6	15
(5)	How can business decision-making tools consider the effects of complex nexus interactions on costs, welfare and ecosystems whilst also including differing temporal and spatial scales of impacts and dependencies?	Group 9	14
(6)	What are the pricing mechanisms that enable nexus resources to be most sustainably managed including comparison of costs of avoiding, mitigating or compensating negative impacts?	Group 6	13
(7)	How can financial institutions effectively internalise the nexus into their routine risk analysis and decision-making practices?	Group 12	13
(8)	What reputational risks or opportunities do nexus impacts and dependencies pose to business?	Group 10	13
(9)	How can the impact of primary production globally be quantified and mapped to identify nexus risk hotspots for retailers?	Group 11	13
(10)	How can the role of biodiversity on the supply and interdependence of food, energy and water be measured and assessed to enable improved decision-making?	Group 2	13
(11)	How can complex nexus interactions and uncertain outcomes be communicated such that they can be easily understood and applied by non-experts (customers and the public)?	Group 5	12
(12)	What common metrics can be devised to enable nexus comparisons to be made to help businesses and investors choose priorities and inform decisions?	Group 2	12
(13)	What are the most effective ways in which information arising from increased supply chain transparency can help foster both greater accountability and greater motivation for positive action across the nexus amongst different actors, whilst also protecting against potential negative consequences for business?	Group 3	12
(14)	What are the links and subsequent strategic opportunities between public health costs and managing food, energy and water systems more sustainably?	Group 6	11
(15)	What geographic scales of decision-making and governance are best suited to address nexus issues, given differing interactions across landscapes and stakeholders?	Group 4	11
(16)	What are the ways in which business models could be changed to incorporate nexus concerns about over consumption and waste?	Group 3	11
(17)	How does the lack of food crop diversity (dominance of wheat-maize-rice) impact upon the sustainability of the food-energy-water-environment nexus and what are the risks to business?	Group 10	11
(18)	What types of policy tools are best suited to positively influencing complex interactions and connections between nexus elements?	Group 4	10
(19)	How can the regulatory system (voluntary and legislative) be amended to reflect potential mismatch in temporal scales that exist between business, political, regulatory and natural cycles?	Group 4	10
(20)	How can the understanding of the nexus of interactions between food, energy, water and the environment be improved to identify specific incentives that either encourage or impede businesses to implement circular economies?	Group 7	10
(21)	Under what conditions do actions that improve the sustainable management of food, energy, water and the environment also enhance long-term business resilience and profitability?	Group 9	10
(22)	What market-based and other financial instruments (including trading systems) will be required to sustain investments in projects designed to achieve sustainable food chains in a volatile world?	Group 9	10
(23)	How does sustainable management of the nexus relate to the resilience of procurement in a world of more unpredictable prices?	Group 1	10
(24)	How does managing outcomes across all four nexus elements influence risk in supply chains?	Group 9	10
(25)	How is the supply and availability of food, energy and water being affected as a result of spatial demographic change and increased competition for land resulting, for example, from urbanisation?	Group 11	10
(26)	How can businesses be incentivised to make investments that will reduce their impacts and create more sustainable dependencies upon food, energy, water and the environment?	Group 12	9
(27)	How can public sector procurement be better harnessed to support business practice that minimises negative impacts across the nexus?	Group 4	8
(28)	How should nexus interactions be incorporated into models to inform decision making for locating new infrastructure, manufacturing sites and technology?	Group 7	8
(29)	How can stakeholders be enabled to work together on a landscape level and beyond individual value chains to best address nexus risks and opportunities?	Group 8	7
(30)	How can the challenges of managing the nexus be integrated into regional/national investment planning?	Group 9	7
(31)	What are the perceptions of the roles of public, private and civil society responsibility in terms of managing natural resources more sustainably, and how can these perceptions be managed or changed to scale up positive action?	Group 2	7
(32)	How can governments support and promote more transparent sustainability reporting by businesses?	Group 4	7
(33)	What are the drivers and barriers that affect private sector decisions to invest in innovative solutions (including technologies) that can have cross-sectoral nexus benefits?	Group 3	7
(34)	How can business leaders be motivated to improve knowledge and action on nexus dependencies and impacts?	Group 9	7
(35)	How can funds and resources be directed into reconfiguring supply chains to integrate more sustainable technologies, management processes and materials?	Group 12	7
(36)	What are the energy and food implications of peak phosphorus as a critical yet finite natural resource?	Group 10	7
(37)	What are the mechanisms to enhance food, water and energy management and production for urban environments so that these are more accessible, equitable and affordable (for both the developed and developing world)?	Group 3	7
(38)	How can behavioural change be enabled, including through the use of financial instruments, to improve stakeholder cooperation to deal with relationships between ecosystem services at a landscape level?	Group 11	6
(39)	How can best practice regarding businesses’ sustainable use or production of food, energy, water and the environment be adapted to accommodate different geographies and cultural settings, that are characterised by distinct operational conditions and priorities?	Group 8	5
(40)	What are the local and global impacts of urban food production on more sustainable management of the nexus and can these be translated into sustainable business opportunities?	Group 11	4

### Questions for business

*Tools for decision*-*making:* There is enormous potential for research and business communities to work together to apply new data and analyses to improve private-sector decision-making (Kareiva et al. 2015). This is underscored by the particular focus on tools for decision-making in the list of prioritised questions: seven questions explicitly dealt with how to effectively incorporate nexus interactions (and their complexity) into decision-making processes (Table 4: Q1, Q5, Q7, Q10–12, Q28). This is indicative of the requirement that business has for systematic and credible methods that can be readily applied; including in the conversion of complex concepts, like the nexus, into clear frameworks or tools that can inform strategic decisions and actions.

The nexus is still a relatively new concept although the recognition that decisions are complex and require multiple trade-offs is not. It will take time for it to integrate into the thinking of sustainability researchers and practitioners in private, public and civil society sectors. Hydrologists and biodiversity specialists, for example, often do not collaborate in shared programmes. Further, organisations (such as NGOs and research institutions) tend to specialise on one or a few issues. The environmental agenda has a dearth of social scientists and a much more interdisciplinary approach is required across the board (Stirling 2015). Almost half of the questions that were submitted did not explicitly mention food, energy, water, or the environment, highlighting the difficulties of comprehending and taking appropriate action on complex and uncertain interactions between ecological and social systems at multiple scales that are inherent to sustainability science, and emphasised in nexus approaches (Kates et al. 2000; Swart et al. 2004). Whilst science increasingly informs many areas of policy and decision-making, it remains a major challenge to develop credible yet accessible tools to help corporate decision-makers understand how a change or shock to one part of the nexus interacts with other domains of the nexus to affect environmental, social and economic systems. The spatial nature of sustainable nexus management is another major challenge for decision-makers: identifying nexus risk hotspots (Q2, Q9), identifying how spatial demographic change can drive nexus resource availability (Q25), helping companies to determine the best locations for siting manufacturing infrastructure (Q28) and even how a shift to urban agriculture may impact upon nexus resources (Q40). There is a wealth of opportunities to share knowledge or expertise and both researchers and practitioners have a vital role in ensuring that opportunities to translate academic research to practical guidance or tools are maximised.

*The commercial case around risk, investments and profitability:* A primary concern that emerged prominently in the top-ranked questions was the need to examine the business case for increased sustainability of nexus resource use. Understanding how risks manifest around unsustainable management of food, energy, water and environmental systems is key for businesses operating under conditions of increased demand for natural resources (Q1–2, Q8–9, Q17, Q23–24, Q29). This includes issues such as how businesses can identify risks (including threats to reputation), demonstrating links between management of the nexus and supply chain security or price volatility, and how collaboration or cooperation can be enabled to address landscape level risks under situations of shared ownership and common goods. Risk is of fundamental importance to investors and this also emerged as a key concern in developing tools and metrics that allow financial institutions to incorporate concerns over nexus resources into their investment decisions (Q7, Q12).

Understanding the conditions under which actions to enhance the management of food, energy, water and the environment contribute to company profits (economic sustainability) is vital to deciding when commercial drivers are sufficient to drive positive change versus situations where government regulation or further incentives will be required (Q21). It is not always in the interest of many businesses to encourage a reduction in consumption. Fundamental, then, is whether there are particular business models and particular conditions or contexts in which overconsumption is decoupled from growth and profits, such that commercial drivers enable businesses to benefit from a reduction in unnecessary natural resource depletion (Q16).

### Questions about business

*Levers for behaviour change:* Awareness and education about sustainability issues from a young age will have profound impacts on how society understands and responds to future challenges and goals (Davis 2009; Jones et al. 2012). However, the relative importance of information and awareness versus personal value systems, remains a critical issue for determining the most effective levers of change for pro-sustainability behaviour amongst decision makers (Hansen et al. 2003). One of the priorities that emerged clearly from this exercise was around *how*, once a particular course of action is identified for more sustainable management of the nexus, businesses or consumers can be encouraged to change their behaviour. The research questions that address this issue will particularly benefit from a multidisciplinary approach, including disciplines such as psychology, sociology, economics and political science, and the natural sciences.

Fundamentally, an understanding is required of the roles and responsibilities of government, businesses and civil society to determine where interventions should be directed and which group of actors stand to be influenced by the research (Q31). High priority questions in this area also focused on identifying the most effective types or classes of intervention; investigating, for example, the contribution of information, pricing, nudging and taxation on business and consumer behaviour (Q3). Likewise, several questions attempted to identify mechanisms by which businesses are incentivised to implement circular economies (Q20) and how incentives or financial instruments might be designed to enable investments in a volatile world (Q22, Q26).

Closely aligned with understanding what motivates, enables or impedes businesses from managing the food-energy-water-environment nexus more sustainably are questions about the valuation of ecosystem services. Two questions in the top ten addressed the most effective ways to incentivise or regulate business to ensure that they recognise the value of ecosystems and manage the services that they provide more sustainably (Q4, Q6). If businesses were to increasingly value the environment by considering their dependencies upon it then there is an expectation amongst some that the commercial driver for sustainability will be recognised and acted upon, at least when that driver is shown to be positive. Equally, policy-makers need to be aware of businesses’ material impacts on resources and how these affect society more widely; this can help them to determine incentives and regulatory levers to change or mitigate negative impacts that affect the nexus.

*Governance and collaboration:* Complex interactions between private and public goods highlight the importance of cooperation between the private and public sectors. A single business operating alone is unlikely to be motivated or able, under competitive market forces, to achieve sustainable management of nexus interactions. Effective governance and collaboration are therefore imperative. Three questions in the top half of the ranked priorities addressed the incorporation of nexus complexity into policy tools and governance systems; this demonstrates that these are key areas for future research. Specifically, the questions in this category focussed on: understanding what scales of governance are best suited to managing the nexus (Q15); developing policy tools capable of dealing with the complexity of interaction between important political agendas (Q18); and developing regulatory systems able to address the multiple and very different timescales of political, regulatory, natural and business cycles (Q19). A further two questions related to enabling collaboration between stakeholders at a landscape level (Q29, Q38). Such collaboration and engagement between actors is absolutely essential in managing nexus issues. More effective collaboration needs both research to investigate the most constructive processes for engagement as well as investment within the private sector to provide employees with the requisite skills and management frameworks to facilitate successful collaboration (e.g., Kingfisher 2015; M&S 2015).

### Leadership and the implementation gap

Leadership is crucial. Assessing the ways in which key individuals within businesses can be motivated to improve knowledge within their business is vital to ensuring that nexus considerations are ‘mainstreamed’ into decision-making processes (Q34). Equally, investigating the factors that encourage particular businesses to take a leadership role that will have wider cross-sectoral benefits will also help practitioners identify mechanisms to scale up sustainable actions (Q33). However, exemplary leadership may not be enough when the challenges facing businesses are specific to the geography, politics and culture of the location of business operations. Therefore, identifying how best practice can be adapted to different geographies also emerged as important (Q39). Leadership from government is also essential, both in how governments can support improvements within the private sector (Q32) but also in how they can harness the enormous buying power of their direct procurement by supporting businesses and supply chains that optimise the sustainable use of nexus resources (Q27).

### Knowledge transfer challenges between research and business

Whilst many subject areas around which researchers and practitioners can collaborate to generate action-oriented research outputs were identified, a significant proportion of the submitted questions were assessed as already sufficiently well understood from a research perspective. This may, therefore, point to inadequate or slow communication of research results rather than inadequate research findings. It highlights again the importance of disseminating results effectively, quickly and widely to achieve maximum impact. Poor dissemination of research findings has been implicated in the research-implementation gap that has been identified in conservation science and other fields, such as health (Crosswaite and Curtice 1994; Knight et al. 2008).

### Concluding remarks

Research questions are rarely sought jointly from practitioners and researchers (Knight et al. 2008; Lang et al. 2012; Sugiyama et al. 2016; Wiek et al. 2012). Funders are increasingly interested in science that can demonstrate a positive impact (SEP 2016). Research agendas co-designed by practitioners and academics can enhance their real-world relevance and already funders are engaging with our results to help inform their strategic priorities. Businesses are also developing research collaborations around some of the identified priorities and are looking at how these might affect stakeholders, including consumers, competitors and regulators.

The translation of research into transformative change is impeded because those best placed to judge the ‘actionable’ nature of the work are too often excluded from the project formulation stage (Lang et al. 2012: Wiek et al. 2012). The next steps from this exercise are for multi-disciplinary panels of expert research scientists and practitioners to convene around each of these themes to devise research projects and establish means of answering these questions. Accordingly, the process of bringing the research and business communities together to develop an updated list of research priorities should be repeated regularly to establish an ongoing and iterative exchange of ideas and needs as new knowledge gaps become apparent and others close.
